# Nanodelivery of essential oils as efficient tools against antimicrobial resistance: a review of the type and physical-chemical properties of the delivery systems and applications

**DOI:** 10.1080/10717544.2022.2056663

**Published:** 2022-04-01

**Authors:** Victoria Dupuis, Constantin Cerbu, Lucjan Witkowski, Adrian-Valentin Potarniche, Maria Cristina Timar, Monika Żychska, Cristina M. Sabliov

**Affiliations:** a Department of Infectious Diseases, Faculty of Veterinary Medicine, University of Agricultural Sciences and Veterinary Medicine Cluj-Napoca, Cluj-Napoca, Romania; b Laboratory of Veterinary Epidemiology and Economic, Institute of Veterinary Medicine, Warsaw University of Life Sciences (SGGW), Warsaw, Poland; c Faculty of Furniture Design and Wood Engineering, Department of Wood Processing and Wood Products Design, Transilvania University of Brasov, Brasov, Romania; d Biological and Agricultural Engineering Department, Louisiana State University and LSU Agricultural Center, Baton Rouge, LA, USA

**Keywords:** Nanodelivery, essential oils, therapy, antibiotic resistance

## Abstract

This review provides a synthesis of the last ten years of research on nanodelivery systems used for the delivery of essential oils (EOs), as well as their potential as a viable alternative to antibiotics in human and veterinary therapy. The use of essential oils alone in therapy is not always possible due to several limitations but nanodelivery systems seem to be able to overcome these issues. The choice of the essential oil, as well as the choice of the nanodelivery system influences the therapeutic efficacy obtained. While several studies on the characterization of EOs exist, this review assesses the characteristics of the nanomaterials used for the delivery of essential oils, as well as impact on the functionality of nanodelivered essential oils, and successful applications. Two classes of delivery systems stand out: polymeric nanoparticles (NPs) including chitosan, cellulose, zein, sodium alginate, and poly(lactic-co-glycolic) acid (PLGA), and lipidic NPs including nanostructured lipid carriers, solid lipid NPs, nanoemulsions, liposomes, and niosomes. While the advantages and disadvantages of these delivery systems and information on stability, release, and efficacy of the nanodelivered EOs are covered in the literature as presented in this review, essential information, such as the speed of emergence of a potential bacteria resistance to these new systems, or dosages for each type of infection and for each animal species or humans is still missing today. Therefore, more quantitative and *in vivo* studies should be conducted before the adoption of EOs loaded NPs as an alternative to antibiotics, where appropriate.

## 1. Introduction

In the 21st century, antibiotic resistance has been identified as one of the world's greatest threats to human and animal health and proved to be a real scourge in the fight against pathogenic microorganisms. This phenomenon is responsible for around 25,000 deaths annually which could become one of the leading causes of death worldwide (Manus, 2019). Today, even the most common infections are becoming difficult to treat. For example, the resistance of *Escherichia coli* to 3rd generation cephalosporins has increased six-fold in healthcare facilities in recent years (Manus, 2019). The massive and unreasonable use of antibiotics has accelerated antibiotic resistance (Jamil et al., 2016) and since the late 1960s, very few new classes of antibiotics have been synthesized, which has highlighted the antibiotic resistance phenomenon (Sabtu et al., 2015). Bacteria such as *Pseudomonas aeruginosa*, *Staphylococcus aureus*, coagulase-negative *Staphylococcus*, *Salmonella* spp., *Shigella* spp., *Enterococcus* spp., and *Escherichia coli* are currently in the spotlight, as being the most antibiotic-resistant bacteria and leading to the most serious infections in humans and animals (Chouhan et al., 2017). The possibility of antibiotic resistance transfer from intestinal bacteria of animals to human via the consumption of foods of animal origin is of concern (Ambrosio et al., 2017) and calls for alternatives to antibiotics in animal production that would be equal or more effective than antibiotics, and could be used as a treatment, during systemic or localized infections, with minimal risk to human and animal health (Cerbu et al., 2021).

Medicinal and aromatic plants have been known for thousands of years for their therapeutic virtues. Their by-products, specific essential oils (EOs) with antimicrobial properties in particular, have been discovered and extensively characterized (Chouhan et al., 2017). However, due to the fact that these extracts are not stable when exposed to various factors such as heat, humidity, oxygen, or light, their direct use has been extremely difficult in human and veterinary therapy (Shetta et al., 2019; Gündel et al., 2020).

In terms of pharmacokinetics, even though EOs benefit from a fast absorption following oral, pulmonary and dermal administration, EOs are quickly metabolized, leading to a short half-life and low bioavailability (Baptista-Silva et al., 2020). For example, it has been demonstrated that the highest level of EOs occurred 2 hours after the administration, with no EOs detected in the bloodstream in just 5 hours (Horky et al., 2019). Interactions with other food components were shown to enhance EOs bioavailability (Horky et al., 2019), and specific formulations can be developed to achieve the same objective. The ultimate goal of using a delivery system for EOs is to improve their delivery, release, and bioavailability in tissues and cells (Baptista-Silva et al., 2020) .

Nanoparticles containing essential oils (EOs NPs) are one of the proposed solutions to fight against the phenomena of antibiotic resistance as various studies carried out over the past ten years show that they could effective in systemic infections (Shin et al., 2019), and localized infections (Saporito et al., 2018). Different delivery systems loaded with entrapped EOs have been developed and when tested *in vitro* (Ghodrati et al., 2019; Sugumar et al., 2015) and *in vivo* (Alam et al., 2018) they proved to improve efficacy of EOs as antimicrobials. Moreover, nanodelivered EOs incorporated directly in animal food proved to be effective in reducing the rate of food-borne infections in animals, in particular because of bacteria (Nouri, 2019; Amiri et al., 2020; Hosseini & Meimandipour, 2018).

A robust literature is available on nanodelivery of essential oils. The type of EOs selected and their antimicrobial properties, as well as the type of nanodelivery systems used to deliver the EOs impact the therapeutic efficacy of the nanodelivered EOs. Therefore, special emphasis is placed in this review on materials used to make the nanodelivery systems, divided into two classes, polymeric and lipidic NPs. The properties of these delivery systems as well as their applications in veterinary and human medicine are also covered. This review contains three main sections: (i) advantages offered by nanodelivery of EOs as antimicrobials, (ii) importance of the characteristics of nanodelivery systems in the delivery of EOs, and (iii) types of nanodelivery systems for EO delivery. The 3^rd^ section also highlights the most important characteristics and pharmacokinetic benefits offered by different delivery systems, together with potential applications and drawbacks. To the best of authors’ knowledge, this is the first review that critically assesses the characteristics of the nanodelivery system itself and impact on stability, release, and functionality of EOs, for efficient and safe delivery of EOs as antimicrobials.

## 2. Advantages offered by nanodelivery of essential oils as antimicrobials

Some studies have shown that EOs are good therapeutic alternatives to antibiotics, to fight against local infections, for example in chronic wounds (Rozman et al., 2020), and against systemic infections, particularly in intensive farming systems where the microbial pressure is often higher than in extensive farming systems (Nouri, 2019). However, the use of free essential oils in food systems or therapy has several limitations, such as (a) high volatility (Shetta et al., 2019; Ghodrati et al., 2019; Nouri, 2019; Almeida et al., 2019); (b) unfavorable effects on organoleptic characteristics (Hosseini & Meimandipour, 2018; Scandorieiro et al., 2016), especially given their strong odor which is confusing for animals when EOs are added to their diet (Hosseini & Meimandipour, 2018; Shetta et al., 2019), and (c) the presence of adverse reactions that decrease both palatability and food intake (Nouri, 2019; Hosseini & Meimandipour, 2018). These reactions result in degradation of essential oils when exposed to various external factors (Ghodrati et al., 2019) such as heat (Shetta et al., 2019; Luis et al., 2020), light, oxygen, pH, humidity (Shetta et al., 2019; Bazana et al., 2019), chemicals (Shetta et al., 2019), pressure (Shetta et al., 2019; Mohammadi et al., 2020), and gastric digestion (Bazana et al., 2019). Other factors such as low solubility (Shetta et al., 2019; Luis et al., 2020), hydrophobic nature (Shetta et al., 2019; Nouri, 2019), low stability especially oxidative instability, low bioavailability (Nouri, 2019), high lipophilicity and poor membrane permeability (Ghodrati et al., 2019) could also be responsible for the failure of EOs to provide good results *in vivo*. Most of these limitations can be overcome by the nanodelivery of essential oils (Hosseini & Meimandipour, 2018; Luis et al., 2020).

The principle of nanoencapsulation/nanoentrapment is fundamental to overcome the limits imposed by the use of free essential oils in therapy. Encapsulation is a process that consists of loading materials within the empty core surrounded by a wall material of a capsule, which allows the protection and controlled release of bioactive compounds (Ezhilarasi et al., 2013). Alternatively, nanoentrapment refers to the loading of a bioactive compound by embedding it into the nanoparticle matrix. Both types of nanodelivery systems can mask the EOs’ unpleasant odor, control their release, increase their solubility and stability, or have an intrinsic antimicrobial effect (Bazana et al., 2019). Nanodelivery not only allows to protect the bioactive compounds of essential oils from the degradation that could occur by direct contact with different environmental factors (light, heat, pH, humidity, oxygen) (Hosseini & Meimandipour, 2018), but it also helps increase their effectiveness (Luis et al., 2020). It has been shown that loading essential oils into nanoparticles can increase their affinity for targets, improve their penetration, and speed up their accumulation process in different cell types (Ghodrati et al., 2019). This allows the active substances to act at the site of interest, increases their ability to remain in the bloodstream for long periods, and protects the active substance from enzymatic hydrolysis (Souza et al., 2017).

Thus, there is an interest in developing new materials at the nanometric scale, which seem promising to improve the delivery and efficacy of EOs (Shetta et al., 2019).

## 3. Importance of the characteristics of nanodelivery systems in the release of essential oils

It is known that the administration of any bioactive compound to various sites in the body is intimately related to the composition, size, surface charge of the nanodelivery system used, along with other factors (Suganya & Anuradha, 2017; Ezhilarasi et al., 2013). The small size of nanoparticles allows them to pass through different biological barriers in order to deliver drugs to various levels (Wang et al., 2020). Relative to microencapsulation (Suganya & Anuradha, 2017; Paulo and Santos, 2017; Mohammadi Gheisar et al., 2015; Castro-Rosas et al., 2017; Sun et al., 2019; Kujur et al., 2017), nanoencapsulation has been shown to have greater potential in terms of bioavailability, controlled release, and precision targeting of bioactive compounds (Suganya & Anuradha, 2017; Ezhilarasi et al., 2013). Indeed, the smaller the size of a particle, the greater the specific surface, the reactivity and the bioavailability of the entrapped drug, resulting in an enhanced functionality of the bioactive, such as antimicrobial efficacy (Basavegowda et al., 2020; Ezhilarasi et al., 2013).

Nanoparticles can be developed into release systems, intended to release the active substance only after it has arrived at the site of action in the body (Suganya & Anuradha, 2017; Ezhilarasi et al., 2013). They can be customized into timed release systems, of a specific release rate of the active substance (Suganya & Anuradha, 2017; Bazana et al., 2019) and their content can be released at controlled rates under specific conditions (Amiri et al., 2020). Several studies report on nanodelivery systems engineered to release the active substance slowly in the body thanks to the encapsulating material (Suganya & Anuradha, 2017). Nanoparticles are protective release systems, capable of shielding the active substance from degradation by external factors (Suganya & Anuradha, 2017; Amiri et al., 2020; Bazana et al., 2019).

Apart from the size, other characteristics seem of paramount importance for the proper functioning of the nanodelivery systems. Among these, the polydispersity index (PDI) is a parameter that is used to assess the particle size uniformity. A PDI lower than 0.3 ensures a size distribution in a colloidal system without the formation of precipitant (Paula Zapelini de Melo et al., 2019). The PDI is an important parameter to measure given that a monodisperse system is able to deliver a consistent amount of compound (PDI < 0.1) in comparison with a polydisperse system (PDI > 0.1) (Gomes et al., 2011; Hill et al., 2013).

Another important parameter is zeta potential (ZP). ZP is not only a good stability indicator of the nanoparticles in suspension as a result of the magnitude of electrostatic repulsion/attraction between particles (Bagheri et al., 2021; Hadidi et al., 2020; Jamil et al., 2016; Paula Zapelini de Melo et al., 2019; Khezri et al., 2020), but also a measurement of nanoparticle interaction with biological systems. Given that the bacterial cell wall carries a negative charge, positively charged NPs will interact stronger with these cells (Jamil et al., 2016; Cinteza et al., 2018).

## 4. Types of nanodelivery systems for EO delivery

Several types of nanodelivery systems have been highlighted in the literature during the last ten years. Among these, we identify two main categories: polymeric and lipidic; which differ from each other by virtue of their characteristics, advantages, drawbacks and applications, but they both have biodegradable and eco-friendly characteristics.

**Figure 1.**
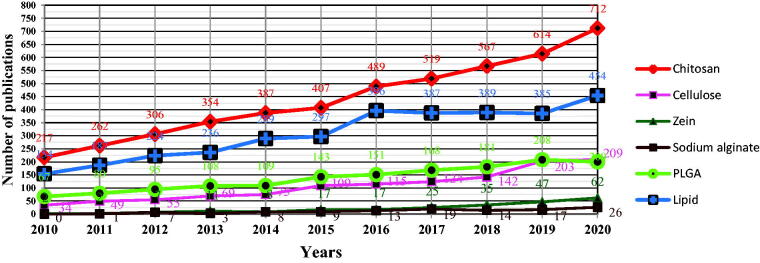
The dynamics of publications for biodegradable and eco-friendly NPs (the ISI Web of Science Core Collection Clarivate Analytics was searched with the following keywords: ‘chitosan’ or ‘cellulose’ or ‘zein’ or ‘sodium alginate’ or ‘PLGA’ or ‘lipid’ and ‘nanoparticles’ (searches carried out on 29/05/2021).

Assessing the dynamics of publications for biodegradable and eco-friendly NPs, made from chitosan, cellulose, zein, sodium alginate, PLGA and lipids (Figure 1), it is apparent that the number of publications gradually increased between 2012 and 2020 for all types of delivery systems. Chitosan NPs stand out with the highest number of hits with a steep and sustained rate of increase over time. During the past 40 years, an important number of papers have been published on chitosan and its potential use in various applications. According to the *Web of Science* database, chitosan as such has been the subject of more than 66000 publications in the past 10 years. Among these publications, more than 4600 have described its use in the field of nanosciences and nanotechnologies. Thus, chitosan seems to be of tremendous importance and has a growing potential as a nanocarrier agent for its use both in food systems and in therapy. Moreover, half of these publications are from the last 4 years (Web of Science, searches carried out on 08/01/2021).

Lipid NPs come second, reaching a plateau between 2016 and 2019, followed by an significant increase. PLGA NPs and cellulose NPs respectively generally seem to follow the same trend. Finally, a lower number of publications mention zein NPs and sodium alginate NPs, with a slowly growing number of publications over the past 10 years.

Of the many scientific publications available on various nanodelivery systems (Figure 1), only a minority describe specifically the use of these nanodelivery systems for delivery of EOs as antimicrobials in medical or veterinary therapy, summarized below.

### 4.1. Natural and eco-friendly materials

#### 4.1.1. Chitosan nanoparticles (CS NPs)

Usually, when talking about chitosan, we refer to polymers that are characterized by the number of sugar units per polymer molecule, which defines the molecular weight and the degree of deacetylation – an important parameter to take into account because it affects the solubility of chitosan in aqueous solutions, so it can also influence the EOs release (Dodane & Vilivalam, 1998).

A substantial number of studies report on EO delivery using chitosan nanoparticles (Table 1). EOs loaded into CS NPs such as Green tea (*Camellia sinensis*) EO (Shetta et al., 2019), Nettle (*Urtica dioica L*) EO (Bagheri et al., 2021), or Clove (*Eugenia caryophyllata*) EO (Hadidi et al., 2020) have been shown to have antibacterial properties against Gram-positive and Gram-negative bacteria, relevant for many systemic or localized infections in humans, companion animals as well as in the production animals. Other EOs loaded into CS NPs are considered to be a suitable alternative to synthetic antibiotic growth promoters (still used in some parts of the world), as in-feed in poultry production with targeted antibacterial activity against pathogenic bacteria, while preserving the development of bacteria of the intestinal flora. Some examples include Thyme (*Thymus vulgaris*) EO (Hosseini & Meimandipour, 2018), Mint (*Mentha piperita*) EO (Nouri, 2019) or Garlic (*Allium sativum*) EO (Amiri et al., 2020). It is hence concluded that EOs loaded into CS NPs are a key player for both medical and veterinary applications.

**Table 1.** CS-EOs NPs characteristics and potential applications.

Characteristics of CS used	EOs loaded into CS NPs	NPs size (nm)	NPs polydispersity index	NPs zeta potential (mV)	Methods used	Functionality	Type of study	Medical or veterinary applications	References
Medium molecular weight (mw) chitosan	Mint (*Mentha piperita*), Thyme (*Thymus vulgaris*), *Cinnamon* (*Cinnamomum verum*)	40–100	Not reported	Not reported	Ionic gelation	Nanoencapsulation improves body weight gain, feed conversion ratio and feed intake on broiler chickens	*In vitro* and *in vivo*	A suitable alternative to synthetic antibiotic growth promoter used as in-feed in poultry production thanks to antibacterial activity against pathogenic bacteria (*Escherichia coli*), while preserving the bacteria of the intestinal flora, such as *Lactobacillus* spp.	(Nouri, 2019)
Medium mw chitosan, 75–85% degree of deacetylation	Cardamom (*Elettaria cardamomum*)	50–100	Not reported	> +50	Ionic gelation	Encapsulation efficiency of more than 90%. Long term stability. Extension of antimicrobial potential up to 7 days compared to 2 days with CSNPs alone	*In vitro*	Antimicrobial potential against extended-spectrum β-lactamase producing *Escherichia coli* and methicillin-resistant *Staphylococcus aureus*	(Jamil et al., 2016)
Medium mw chitosan	*Homalomena pineodora*	70	0.176	> +24	Ionic gelation	High encapsulation efficiency and loading capacity. Initial burst release followed by a slower release, up to complete release at 72 h. Release profile controlled by the first order kinetic model. Concentration-dependent killing behavior on time–kill assay	*In vitro*	Antimicrobial activity broad-spectrum against diabetic wound pathogens: *Bacillus cereus*, *Bacillus subtilis*, *Staphylococcus aureus,* and methicillin-resistant *Staphylococcus aureus* (Gram+). *Escherichia coli*, *Proteus mirabilis*, *Yersinia* spp., *Klebsiella pneumoniae*, *Shigella boydii*, *Salmonella typhimurium*, *Acinetobacter anitratus* and *Pseudomonas aeruginosa* (Gram-)	(Rozman et al., 2020)
Not reported	Garlic *(Allium sativum*)	Not reported	Not reported	Not reported	Ionic gelation	Nanoencapsulation improves body weight gain, feed conversion ratio and feed intake on broiler chickens	*In vitro* and *in vivo*	A suitable alternative to synthetic antibiotic growth promoter used as in-feed in broiler production thanks to antibacterial activity against *Escherichia coli*	(Amiri et al., 2020)
Medium mw chitosan (684 kDa), Roughly 85 % degree of deacetylation	Cinnamon (*Cinnamomum zeylanicum*)	100–200	<1	> +38	Ionic gelation	Initial burst release in the first 9 days, followed by a slow release. Release faster at low pH. Release profile follows a Fickian behavior	*In vitro*	Antibacterial activity against *Escherichia coli*, *Erwinia carotovora*, and *Pseudomonas fluorescens* (Gram-)	(Mohammadi et al., 2020)
Medium mw chitosan	Thyme *(Thymus vulgaris*)	30–100	Not reported	Not reported	Ionotropic gelation	Nanoencapsulation improves body weight gain and feed conversion ratio on broiler chickens. Initial burst release (97%) in the first 96 hours, followed by a slower release	*In vitro* and *in vivo*	A suitable alternative to synthetic antibiotic growth promoter used as in-feed in poultry production thanks to antibacterial activity against pathogenic bacteria (coliforms, aerobes), while preserving the bacteria of the intestinal flora, such as *Lactobacillus* spp.	(Hosseini and Meimandipour, 2018)
Medium mw chitosan 75–85% degree of deacetylation	Rosemary (*Rosmarinus officinalis)* Oregano (*Origanum vulgare* subsp. *hirtum)* Lavender (*Lavandula angustifolia*) Marine criste (*Crithmum maritimum)* White fir *(Abies alba*) Wild chamomile (*Matricaria chamomilla*) Pennyroyal (*Mentha pulegium)* Sage (*Salvia officinalis)* Anise (*Pimpinella anisum)*	250–300 and 500–600	Not reported	Not reported	Ionotropic gelation	Initial burst release followed by a slower release reaching a plateau	*In vitro*	Antibacterial activity against *Staphylococcus aureus*, *Bacillus subtilis*, *Bacillus cereus* (Gram+) and *Escherichia coli*, *Xanthomonas campestris* (Gram)	(Halevas et al., 2017)
Medium mw chitosan, 84.8% degree of dealkylation	Peppermint (*Mentha piperita*) Green Tea (*Camellia sinensis*)	20–60	Not reported	+20–+23and +24–+29	Emulsification/ionic gelation	Thermal stability of EOs-CS NPs reaching 350 °C. Initial burst release in the first 12 h, followed by a slower release up to 72 h. Release faster at low pH. Release profile follows a Fickian behavior	*In vitro*	Antibacterial activity against *Staphyloccocus aureus* (Gram+) and *Escherichia coli* (Gram-)	(Shetta et al., 2019)
Low mw chitosan (50–190 kDa), 80% degree of deacetylation	Nettle (*Urtica dioica L*)	208–369	0.153–0.412	+14–+30	Emulsion-ionic gelation in two stages: oil-in-water emulsification and then, ionic gelation	Not reported	Not reported	Antibacterial activity against *Staphylococcus aureus*, *Bacillus cereus*, *Listeria monocytogenes* (Gram+) and *Escherichia coli*, *Salmonella typhi* (Gram-)	(Bagheri et al., 2021)
Low mw chitosan (50–190 kDa), 75–85 % degree of deacetylation	Clove *(Eugenia caryophyllata*)	223–445	0.117–0.337	+10–+34	Emulsion-ionic gelation in two stages: oil-in-water emulsification and then, ionic gelation	Not reported	*In vitro*	Antibacterial activity against *Staphylococcus aureus*, *Listeria monocytogenes* (Gram+) and *Escherichia coli*, *Salmonella typhi* (Gram-)	(Hadidi et al., 2020)
Medium mw chitosan 75–85% degree of deacetylation	Oregano (*Origanum vulgare*)	282–402	Not reported	Not reported	Oil-in-water emulsion and ionic gelation	Initial burst release followed by a slower release	*In vitro*	Not reported	(Hosseini et al., 2013)
Medium mw chitosan 75–85% degree of deacetylation	Ajwain (*Carum copticum*)	236–721	Not reported	Not reported	Emulsion-ionic gelation	Initial burst effect for the first 24 h, followed by a steady release for 72 h, before decreasing and reaching a plateau. Release faster at low pH	*In vitro*	Antibacterial activity against *Staphylococcus aureus*, *Staphylococcus epidermidis*, *Bacillus cereus* (Gram+) and *Escherichia coli*, *Salmonella typhimurium*, *Proteus vulgaris* (Gram-)	(Esmaeili and Asgari 2015)
Medium mw chitosan 75–85% degree of deacetylation	Thyme (*Thymus vulgaris*)	6	Not reported	Not reported	Nanoprecipitation	Release time between 360 and 390 min	*In vitro*	Antibacterial activity against *Staphylococcus aureus*, *Bacillus cereus*, *Listeria monocytogenes* (Gram+) and *Escherichia coli*, *Salmonella typhi*, *Shigella dysenteriae* (Gram-)	(Sotelo-Boyás et al., 2017)

##### 4.1.1.1. Characteristics of the chitosan NPs and impact on functionality of nanodelivered EOs

Chitosan NPs of different characteristics (monodisperse, ranging from 50 to 600 nm in diameter, positively charged) were developed for delivery of EOs, with different impact on EO functionality (Table 1). A study conducted on Tilapia (*Oreochromis nilotica*) demonstrated that CS NPs may have different metabolic pathways compared to CS and that this allows them to improve digestion and absorption of nutrients at lower EOs inclusions levels (Hosseini & Meimandipour, 2018). CS NPs are able to bind the EOs and open the tight junction in the gut cells, in order to allow a better absorption of EOs (Nouri, 2019). The nanoparticles delivery system is able to transport the essential oil to the surface of the bacterial cell membrane and improve its uptake, while the pure essential oil (with low solubility in water) could not easily be in contact with the cell layers (Mohammadi et al., 2020). Another study has shown that the surface chemistry of CS NPs affects the interaction with the cells in the physiological environment during drug delivery via cutaneous route on chronic wounds (Rozman et al., 2020), while other factors such as the size and shape of nanoparticles also play a fundamental role in the performance of the NPs (Rozman et al., 2020; Hosseini et al., 2013). Several mechanisms, such as surface erosion, disintegration, diffusion, and desorption have been reported to explain how EOs could be released from CS NPs. The main mechanism responsible for the release of EO has been shown to be diffusion of EO out of CS NPs into the external environment, followed by degradation of the polymer matrix. Generally, the rate of release is initially very high, followed by a subsequent slow release of the EO (Esmaeili and Asgari, 2015; Hosseini et al., 2013; Sotelo-Boyás et al., 2017).

Chitosan NPs with controlled release profiles were developed to improve the efficacy of the nanodelivered EOs. Generally, the release profile had an initial burst between 12 hours and 9 days, followed by a slower release (Shetta et al., 2019; Hosseini et al., 2013; Hosseini and Meimandipour, 2018; Mohammadi et al., 2020; Halevas et al., 2017; Esmaeili and Asgari, 2015). The release profile followed a Fickian behavior (Mohammadi et al., 2020; Shetta et al., 2019), or a first order of kinetic model (Rozman et al., 2020). Several studies agreed that the release was faster at low pH (Mohammadi et al., 2020; Shetta et al., 2019; Esmaeili and Asgari, 2015) which could be explained by the swelling and partial dissolution of the CS NPs (Shetta et al., 2019). To increase encapsulation efficiency and loading capacity, the use of a higher weight ratio of EO to chitosan was recommended (Rozman et al., 2020; Hosseini et al., 2013). Besides, efficient encapsulation and release of EOs, CS NPs has proven itself as a suitable EOs delivery system in broiler chickens, improving body weight gain, feed conversion ratio (Nouri, 2019; Amiri et al., 2020; Hosseini and Meimandipour, 2018) and even feed intake (Nouri, 2019; Amiri et al., 2020).

##### 4.1.1.2. Advantages and drawbacks of chitosan nanodelivery systems

Chitosan is considered to be a superior carrier agent due to its polysaccharide nature with mucoadhesive, nontoxic, and renewable properties (Yadav et al., 2020). The higher the molecular weight of chitosan, the better its mucoadhesion, with an ideal value of approximately 1400 kDa (Dodane & Vilivalam, 1998). Chitosan has antimicrobial potential which has been described as being innate (Jamil et al., 2016) – probably related to the reduction of the permeability of bacterial cell membranes thanks to the interaction of positively charged amino groups of chitosan and the negatively charged microbial cells (Hosseini & Meimandipour, 2018; Nouri, 2019). Furthermore, studies indicated that the positively charged nanoparticles were able to alter the electron transport chain of the bacterial membrane (Rozman et al., 2020; Packia Lekshmi et al., 2012), and that chitosan has the ability to form a film, of good permeability, and high tensile strength (Hosseini & Meimandipour, 2018). Chitosan, depending on its molecular weight and its degree of deacetylation has hemostatic properties, thanks to the positive charges that can bind to negative charges of erythrocytes (Yahya et al., 2020; Bano et al., 2017). Moreover, its positive charges improve absorption and provide strong binding to carboxyl-negative charges on bacterial cell walls (Nouri, 2019). Chitosan is the most studied and the most appropriate nanocarrier for the delivery of EOs because of its abundance, its GRAS (= Generally Recognized As Safe) status, its high encapsulation efficiency, and its controlled release of EOs (Chaudhari et al., 2020). It has a low production cost, good biodegradability, good biocompatibility, and its use has therefore drastically increased in recent years in the pharmaceutical and food applications (Wang et al., 2020). Effectively, chitosan-based nanomaterials have been found acceptable for food applications by consumers, and even by the food industry and regulatory agencies (Kalagatur et al., 2018). Besides its own properties described above, CS NPs act synergically with the EOs, improving their thermal stability, protecting their phenolic content, ensuring a prolonged release profile, and improving their antioxidant as well as their antibacterial activity; all of this for nutraceutical, cosmetic and pharmaceutical use (Shetta et al., 2019). No major drawbacks of CS NPs have been described so far, which make it very attractive for several medical or veterinary applications, especially when compared to other types of nanoparticles.

##### 4.1.1.3. Potential applications of CS-EOs NPs

As briefly discussed above, CS NPs loaded with EOs could be used as an alternative to antibiotics to improve animal performance, especially in intensive farming systems. The role of chitosan here is to increase the beneficial effects of EOs, delivered via food (Nouri, 2019; Hosseini & Meimandipour, 2018; Amiri et al., 2020)- it allows more efficient delivery of EOs to the target site in the gastrointestinal tract (Raphaël & Meimandipour, 2017). On the other hand, its unique biological characteristics (hemostatic, broad-spectrum antibacterial, and mucoadhesive properties) allow its use in wound dressing with ideal release behavior intended to cure local infections, to stop hemorrhages, and to support the wound healing process (Yahya et al., 2020; Rozman et al., 2020).

#### 4.1.2. Cellulose nanomaterials

Cellulose is the most abundant biopolymer on our planet and can be obtained from many renewable and sustainable sources such as primary and secondary cell walls of plants, or bacteria and some animals (Yahya et al., 2020). Cellulose is frequently used to carry various types of substances, and particularly EOs, characteristics and applications of which are reported herein (Table 2).

**Table 2.** Cellulose-EOs nanomaterials characteristics and potential applications.

Characteristics of cellulose used	EOs loaded into cellulose nanomaterials	Nanomaterials size (nm)	NPs polydispersity index	NPs zeta potential (mV)	Methods used	Functionality	Type of study	Medical or veterinary applications	References
Cellulose nanocrystals (CNCs)	Thyme white (*Thymus vulgaris*)	Width of 10 and length of 274	Not reported	Not reported	CNCs are produced by hydrolysis of sulfonic acid and used for the formation of the Pickering emulsion with EOs	Not reported	*In vitro*	Antibacterial activity against *Staphylococcus aureus* (Gram+), and *Escherichia coli* (Gram-)	(Shin et al., 2019)
Cellulose nanofibers (CNFs)	Thyme (*Thymus vulgaris*)	Not reported	Not reported	Not reported	CNFs are prepared by enzymatic hydrolysis pretreatment and TEMPO (2, 2, 6, 6-tetram-ethylpiperidine-1-oxide)-mediated oxidation pretreatment	Not reported	*In vitro*	Antibacterial properties were tested through fresh beef experiments, to preserve fresh food from contamination by bacteria	(Zhang et al., 2020)
Cellulose nanofibers (CNFs)	Thyme (*Thymus vulgaris*)	Not reported	Not reported	Not reported	Supercritical impregnation of active molecules, such as EOs, onto nanocellulose three-dimensional (3 D) structures	Not reported	*In vitro*	Antibacterial activity against *Staphylococcus epidermidis* (Gram+), and *Escherichia coli* (Gram-)	(Darpentigny et al., 2020)
Carboxymethyl cellulose (CMC) films	Santolina (*Santolina chamaecyparissus*), Pepper tree (*Schinus molle),* Eucalyptus (*Eucalyptus globulus)*	Not reported	Not reported	Not reported	Preparation of CMC-based films containing EOs	Not reported	*In vitro*	Antibacterial activity against *Staphylococcus aureus*, *Bacillus subtilis*, *Enterococcus faecalis* (Gram+), and *Escherichia coli*, *Pseudomonas aeruginosa*, *Salmonella typhi* (Gram-)	(Simsek et al., 2020)

##### 4.1.2.1. Characteristics of cellulose nanomaterials and impact on functionality of nanodelivered EOs

Cellulose-based aerogel can immobilize or encapsulate inside its cellulose network EOs with antibacterial properties. Moreover, cellulose, both in the form of nanocrystal (CNC) or nanofiber (CNF) benefits from a high surface area, high strength, and adjustable surface chemistry; properties permitting itself to have controlled interactions with other molecules, such as EOs (Yahya et al., 2020). CNCs have a dense shell that allows the reduction in volatility of EOs therefore decreasing the speed of EOs release. Thus, CNCs enable a longer and more sustained antimicrobial activity of EOs in the environment thanks to a controlled release of EOs over the desired period (Shin et al., 2019). CNFs also allow controlled and sustained drug delivery due to their binding to CNFs chains (Pandey, 2021).

##### 4.1.2.2. Advantages and drawbacks of cellulose as nanodelivery systems

Nanocellulose is nontoxic (Bacakova et al., 2019) and nanocellulose materials are known to have no adverse effects on health and the environment (Darpentigny et al., 2020). When used to stabilize Pickering emulsions, nanocellulose with entrapped EOs are more advantageous than other synthetic or inorganic nanoparticles due to the factors such as better biocompatibility, degradability, and lower cost (Shin et al., 2019). Cellulose nanocrystals are considered to be better than cellulose spheres or nanofibers because this allows better control of the morphology and reproducibility of emulsion formation (Shin et al., 2019). Nanocellulose has unique biological characteristics due to its hemostatic, antibacterial, and mucoadhesive properties, which are very useful in healing wounds and stopping bleeding (Yahya et al., 2020). In addition, cellulose’s water absorption and retention capacity are high, which gives it a good drainage capacity of exudates from wounds, while supporting and improving the growth and proliferation of cells (Bacakova et al., 2019).

As drawbacks, we can note that cellulose nanofibers alone, like carboxymethyl cellulose films without EOs (Simsek et al., 2020) do not have any antimicrobial properties (Pandey, 2021), contrary to others such as chitosan nanoparticles which have intrinsic antimicrobial properties. Furthermore, cellulose nanofibers toxicology assessment is under a critical debate given its frequent clinical applications (Pandey, 2021).

##### 4.1.2.3. Potential applications of cellulose-EOs NPs

Currently, limited data is available on the therapeutic applications of cellulose in nanoform (Pandey, 2021; Simsek et al., 2020). For example, cellulose nanomaterials such as crystals are suitable for systemic use and they have antimicrobial action when encapsulating EOs (Shin et al., 2019). Cellulose nanomaterials encapsulating EOs are also suitable for topical use because of their antimicrobial, hemostatic, and mucoadhesive action of use in the treatment of superficial and deep wounds (Darpentigny et al., 2020).

#### 4.1.3. Zein NPs

Zein has been recognized to be a particularly interesting protein for nanoparticles production (Gonçalves da Rosa et al., 2020). It belongs to a family of prolamins which are composed of hydrophobic amino acids (Merino et al., 2019), such as proline, leucine, glutamine and alanine (da Rosa et al., 2015). Zein has been shown to be a promising carrier agent (Table 3) thanks to its biodegradability and biocompatibility, making it a good candidate for the nanodelivery of active substances, and the release of nutrients and drugs (Wu et al., 2012).

**Table 3.** Zein-EOs NPs characteristics and potential applications.

Characteristics of zein used	EOs loaded into zein NPs	NPs size (nm)	NPs polydispersity index	NPs zeta potential (mV)	Methods used	Type of study	Functionality	Medical or veterinary applications	References
Zein from maize, Strongly hydrophobic because of a high proportion of cationic amino acids.	Clove *(Eugenia caryophyllata*) Garlic *(Allium sativum*)	150	<0.2	+30	Antisolvent precipitation, which consists of a hydro-ethanolic zein solution injection into an aqueous solution	*In vitro*	Encapsulation efficiency of more than 90%, stability in storage for 90 days	Applications in aquaculture, thanks to its bactericidal activity against *Streptococcus iniae* (Gram+), *Aeromonas hydrophila*, *Edwardsiella tarda* (Gram-).	(Luis et al., 2020)
Zein from maize	Oregano (*Origanum vulgare*) Thyme (*Thymus vulgaris*)	138–162	0.165–0.191	+20.9–+23.2	Nanoprecipitation with a nonionic Pluronic surfactant	*In situ*	Physical and chemical stability in storage for 90 days at 4 °C and 20 °C, release profile controlled by the Korsmeyer-Peppas kinetic model	Greater antimicrobial activity against Gram + bacteria, such as *Listeria monocytogenes* ATCC 7644 and *Staphylococcus aureus* ATCC 2593 than Gram – bacteria, such as *Escherichia coli* ATCC 25922 and *Salmonella enterica* serovar *Typhimurium* ATCC 14028 (because of their external lipopolysaccharide layer in their membrane which avoids the diffusion of hydrophobic compounds)	(Gonçalves da Rosa et al., 2020)
Zein from maize	Phenolic monoterpenes: Thymol and Carvacrol. There are components of several EOs extracted from Thyme (*Thymus vulgaris*) or Oregano (*Origanum vulgare*)	108–122	0.223 − 0.277	+9–+30	Nanoprecipitation	*In vitro*	Stability in storage for 90 days at 6 °C and 20 °C, release profile controlled by the Korsmeyer-Peppas kinetic model (50% in 72 h without burst effects)	Greater antimicrobial activity against Gram + bacteria, such as *Listeria monocytogenes* ATCC 7644 and *Staphylococcus aureus* ATCC 2593 than Gram– bacteria, such as *Escherichia coli* ATCC 25922 and *Salmonella enterica* serovar *Typhimurium* ATCC 14028	(da Rosa et al., 2015)
Zein from maize	*Ocimum gratissimum* *Pimenta racemosa*	150	<0.3	+16–+32	Nanoprecipitation	*In vitro*	High encapsulation efficiency, physical and chemical stability in storage for 180 days at 6 °C and 20 °C	Promising potential to application in a food system. Can act as natural preservative due to good physicochemical stability during 180 days of storage at 6 and 20 °C	(Paula Zapelini de Melo et al., 2019)
Zein from maize	Thyme (*Thymus capitatus*)	<180	0.250	Not reported	Self-assembly	*In vitro*	Not reported	Bacteriostatic activity improved against Gram + bacteria: *Listeria monocytogenes*, andGram – bacteria: *Escherichia coli* O157:H7	(Merino et al., 2019)
Zein with a minimum protein content of 97 g/100g	Thymol and Carvacrol, essentially found in Thyme (*Thymus vulgaris*) or Oregano (*Origanum vulgare*)	<800	<0.3	Not reported	Liquid-liquid dispersion	*In vitro*	Not reported	Antimicrobial activity against nonpathogenic *Escherichia coli* ATCC 53323 (Gram-). The nonpathogenic bacteria have been chosen because of their similarity to well-known pathogenic bacteria responsible for several foodborne illness outbreaks	(Wu et al., 2012)

##### 4.1.3.1. Characteristics of the zein NPs and impact on functionality of nanodelivered EOs

The most important characteristic of zein is its solubility in alcoholic solutions, due to the high proportion of non-polar amino acids and the deficiency of basic and acidic amino acids (Paula Zapelini de Melo et al., 2019). Zein benefits from GRAS status and it has been classified as a food-grade ingredient by the FDA. Hence, zein was used for drug delivery, vitamin protection in food, as an antioxidant, and emulsifier. It has different morphology/solubility under different pH conditions which makes it versatile and exceptionally suitable for a variety of uses (Wu et al., 2012). Particles range from 100 to 800 nm, some more monodisperse than others (PDI 0.1–0.3 and higher) (Table 2). Different release profiles can be achieved by zein from a continuous release of EOs from the zein NPs during the first 8 h, followed by a slow release without any burst effects (Gonçalves da Rosa et al., 2020), to the rapid release in the presence of bacteria (Wu et al., 2012). Generally, for zein NPs, the EO release profile followed the Korsmeyer-Peppas kinetic model (Gonçalves da Rosa et al., 2020; da Rosa et al., 2015).

It has been demonstrated that zein-EOs NPs are able to improve EOs stability during storage between 90 and 180 days at 4 °C, 6 °C, and 20 °C (Luis et al., 2020; Gonçalves da Rosa et al., 2020; da Rosa et al., 2015; Paula Zapelini de Melo et al., 2019). According to the study from Paula Zapelini de Melo et al. (2019), the use of nonionic surfactant is of paramount importance in order to prevent NPs aggregation and maintain their stability for a longer period of time.

##### 4.1.3.2. Advantages and drawbacks of zein as encapsulating NPs

Thanks to a strong interaction between zein and EOs, zein is a good wall material for EO nanodelivery (Gonçalves da Rosa et al., 2020). Zein has been shown to have a high encapsulation efficiency, and it prevents the degradation of the active compound during storage (for a minimum period of 90 days) (Gonçalves da Rosa et al., 2020). Zein has the ability to form flexible biodegradable films at low cost and resistant hydrophobic coatings which protect against bacteria. It even exerts a protective effect against the toxicity of certain botanical compounds. Moreover, zein NPs containing EOs represent a viable and effective formulation that allows reducing the amount of active substances needed while improving the stability of the natural compounds and maintaining their bactericidal effects (Luis et al., 2020).

An important limitation is the scarcity of toxicological studies that will need to be performed for a better understanding of the effects of zein nanodelivery system (Luis et al., 2020).

##### 4.1.3.3. Potential applications of zein-EOs NPs

Zein-EOs NPs could be used in aquaculture for more sustainable production of fish due to its bactericidal action against pathogenic bacteria in fish where it could replace several antibiotics, such as oxytetracycline, florfenicol, amoxicillin, and erythromycin (Luis et al., 2020). Zein nanodelivery systems could be used as an antimicrobial against both Gram-positive and Gram-negative bacteria, responsible for various types of infections (Merino et al., 2019; da Rosa et al., 2015; Wu et al., 2012).

#### 4.1.4. Sodium alginate

Sodium alginate (NaAlg) is a natural polysaccharide derived from brown algae cell walls, especially *Macrocystis pyrifera*, *Laminaria hyperborea*, and *Ascophyllum nodosum* (Hassani et al., 2020). It contains unbranched chains composed of β-D-Mannuronate and α -L-Glucuronate residues, linked by a β-(1-4) glycosidic covalent bond (Liakos et al., 2014). NaAlg is widely used in synthesis of nanodelivery systems due to its nontoxicity, good biocompatibility, and its ability to be crosslinked. NaAlg films are suitable for encapsulation of bioactive substances in pharmaceutical applications (Hassani et al., 2020), including delivery of EOs (Table 4).

**Table 4.** NaAlg-EOs films characteristics and potential applications.

Characteristics of NaAlg used	EOs loaded intoNaAlg films	NPs size (nm)	NPs polydispersity index	NPs zeta potential (mV)	Methods used	Functionality	Type of study	Medical or veterinary applications	References
Alginic acid sodium salt with viscosity 15000–20000 cps	Elicriso italic *Chamomile* (*Chamomile blue*) *Cinnamon* (*Cannella corteccia*) Lavender (*Lavandula angustifolia*) Tea tree (*Melaleuca alternifolia*) Peppermint (*Mentha piperita*) Eucalyptus(*Eucalyptus globulus)* Lemongrass (*Cymbopogon flexuosus*) Lemon (*Citrus limon*)	-	-	-	Films made of EOs dispersed in NaAlg matrix. With the addition of glycerol to induce plasticity and surfactants to improve the dispersion of the EOs through the NaAlg matrix	Progressive release of the EO from the film for long periods (1–17 days) in a very moist environment	In vitro	Antimicrobial activity against *Escherichia coli* (Gram-)	(Liakos et al., 2014)
Sodium alginate salt with viscosity 15000–20000 cps	Pepper tree (*Schinus terebinthifolius*) *Allspice* (*Pimenta dioica*) Black pepper (*Piper nigrum*)	-	-	-	Films made of EOs dispersed in NaAlg matrix	Release of the EO from the film in a very moist environment	In vitro	Antimicrobial activity against *Staphylococcus aureus* ATCC 29213, *Bacillus cereus* ATCC 11778 (Gram+), and *Escherichia coli* ATCC 35218 (Gram-)	(Rosa et al., 2018)

##### 4.1.4.1. Characteristics of the NaAlg NPs aerogels and impact on functionality of nanodelivered EOs

Aerogel is formed by the replacement of the liquid of a gel by a gas, but without any structural change. The skeletal structure of the aerogels such as those made by NaAlg limits the release of the active substances, and the release rate decreases in time (Qin et al., 2020; Yahya et al., 2020). Control of the pore size is a key element in the amount of substance that is released. The large surface area involves low dissolution properties of the active substances in the aerogel which is important for the long time delivery of the active substances. Hydrophilic aerogels are ensuring a fast active substances release, which makes them an interesting candidate for the controlled release of poorly water-soluble substances such as EOs (Yahya et al., 2020). So far few studies exist on the antimicrobial activity of EOs combined with NaAlg in aerogel form (Table 4), all *in vitro* (Liakos et al., 2014; Rosa et al., 2018). The release of the EO from the films occurred in a very moist environment thanks to the adsorption of humidity by the NaAlg matrix (Rosa et al., 2018) over a period of 1–17 days. In fact, the aerogel did not completely dissolve by the end of the period tested and it retained the EOs, which provides a great advantage in preserving EO’s functionality over time (Liakos et al., 2014).

##### 4.1.4.2. Advantages and drawbacks of NaAlg as encapsulating aerogels and NPs

NaAlg is considered a nontoxic (Hassani et al., 2020), natural, biodegradable, and biocompatible material, which can absorb 200 to 300 times its weight in water. In contact with a humid environment, it can swell and release drugs and encapsulated molecules (Liakos et al., 2014). Its ability to preserve a solid type attribute even under acidic conditions makes it highly attractive for biological applications. It has hemostatic properties, useful in bleeding wounds treatment, and has mucoadhesive properties. It is also able to protect the bioactive compound against physical stress (Yahya et al., 2020).

When compared with the other nanocarriers, one potential drawback would be that NaAlg films have no inherent antimicrobial properties. Thus, colonization of the wound by bacteria prior to EOs release can delay the healing process and lead to potential complications (Liakos et al., 2014).

##### 4.1.4.3. Potential applications of NaAlg-EOs films

NaAlg-EOs films could be used in the production of dressings with good healing properties (Liakos et al., 2014), both in superficial and in chronic wounds (Rosa et al., 2018).

#### 4.1.5. Plga-based NPs

Poly (D, L-lactic-co-glycolic) acid (PLGA) is a copolymer that has been widely used for synthesis of nanoparticles (Sharma et al., 2016). Its hydrolysis leads to metabolite monomers, lactic acid and glycolic acid (Nallamuthu et al., 2013). These two monomers are endogenous and almost effortlessly metabolized by the body via the Krebs cycle; therefore minimal systemic toxicity is reported with the use of PLGA for substance delivery or other biomedical applications (Nallamuthu et al., 2013). PLGA NPs developed for nanodelivery of essential oils were reported to improve their antimicrobial properties (Table 5).

**Table 5.** PLGA-based-EOs NPs characteristics and potential applications.

Characteristics of PLGA used	EOs loaded into PLGA-based NPs	NPs size (nm)	NPs polydispersity index	NPs zeta potential (mV)	Methods used	Functionality	Type of study	Medical or veterinary applications	References
PLGA with a copolymer ratio of DL-lactide to glycolide of 50: 50. PLGA with a molecular weight between 5000–15000 Da	Black caraway (*Nigella sativa*)	148	0.2	−24.8	Solid-in-oil-in-water solvent evaporation	In the 1st 10 h, release of 25% with a burst effect, followed by a sustained release up to 54% for gastric juice and 75% for intestinal juice at 7 days. Better release rate in acidic pH. Physical properties (size, ZP,) of the NPs slightly modified due to heat treatments(60 °C, 80 °C and 100 °C)	*In vitro*	Antimicrobial activity against Staphylococcus aureus (Gram+) and Salmonella typhi, Escherichia coli (Gram-)	(Nallamuthu et al., 2013)
PLGA with a copolymer ratio of DL-lactide to glycolide of 65: 35	Clove *(Eugenia caryophyllata*) Cinnamon (*Cinnamomum* spp.)	200	>0.1	Not reported	Emulsion evaporation	Release with an initial burst effect, followed by a slower and sustained rate. Entrapment efficiency between 92–98%. Release profile controlled by the 2-term exponential kinetic model	*In vitro*	Antimicrobial activity against *Listeria* spp. (Gram+) and *Salmonella* spp. (Gram-)	(Gomes et al., 2011)
PLGA with a copolymer ratio of DL-lactide to glycolide of 50: 50. PLGA with a molecular weight between 38000–54000 Da	Anise (*Pimpinella anisum*) Star anise (*Illicium verum*) Fennel (*Foeniculum vulgare*) Carawa*y* (*Carum carvi*) Dill (*Anethum graveolens*)	126 and 158	0.08–0.2	Not reported	Emulsification solvent evaporation and nanoprecipitation	Release with an initial burst effect during the first 6 h. Controlled release during more than 4 days	*In vitro*	Antimicrobial activity against Staphylococcus aureus (Gram+) and Salmonella typhi, Enterococcus coli (Gram-)	(Esfandyari- Manesh et al., 2013)
PLGA with a copolymer ratio of DL-lactide to glycolide of 65: 35 and 50:50	Cinnamon (*Cinnamomum* spp.)	145–167	0.18–0.26	Not reported	Emulsion evaporation	Release with an initial burst effect during the 1st h but reaching a steady plateau quickly	*In vitro*	Antimicrobial activity against *Listeria monocytogenes* (Gram+) and *Salmonella enterica* serovar Typhimurium (Gram-)	(Hill et al., 2013)
PLGA with a copolymer ratio of DL-lactide to glycolide of 85: 15. PLGA with a molecular weight between 50000–75000 Da	Lemongrass (*Cymbopogon citratus*)	277	0.18	−16	Emulsification/solvent diffusion with Box-Behnken design	Release with an initial burst effect (25% release after 3 h), followed by a sustained release until 84% release after 8 days. Release profile controlled by the Korsmeyer-Peppas model	*In vitro*	Promising potential for pharmaceutical uses, in controlling the release and in reducing the toxicity of the EO	(Almeida et al., 2019)
PLGA with a copolymer ratio of DL-lactide to glycolide of 50: 50	Phenolic monoterpene: Carvacrol. It is a component of several EOs extracted from Thyme (*Thymus vulgaris*) or Oregano (*Origanum vulgare*)	210	0.26	−18.99	Solvent displacement	Release with an initial burst effect (60% release after 3 h), followed by a slower rate until 95% release after 24 h	*In vitro*	Alter the properties of preformed staphylococcal biofilms (*Staphylococcus epidermidis* ATCC 35984)	(Iannitelli et al., 2011)

##### 4.1.5.1. Characteristics of the PLGA-based NPs and impact on functionality of nanodelivered EOs

The release of EOs, and particularly Cinnamon (*Cinnamomum* spp.) and Clove *(Eugenia caryophyllata*) from the PLGA-NPs is generally characterized by an initial burst effect during the 1^st^ hour followed by a steady plateau (Hill et al., 2013; Gomes et al., 2011), in accordance with the modified 2-term Fickian model (Gomes et al., 2011; Danhier et al., 2012). Several factors are involved in the EO release, such as the affinity of EO for the PLGA-NPs, diffusion of active compound through the polymer matrix, polymeric erosion, PLGA swelling, and degradation (Hill et al., 2013). PLGA NPs have a degradation time that can vary from several months to several years, depending mainly on to main characteristics: molecular weight and copolymer ratio (Sharma et al., 2016).

Only in vitro studies have been conducted using PLGA-based-EOs NPs, generally monodisperse, negatively charged and measuring 100–300 nm (Nallamuthu et al., 2013; Gomes et al., 2011; Esfandyari-Manesh et al., 2013). EOs release profile occurred according to a biphasic pattern, with an initial burst effect within 1 and 10 initial hours (Hill et al., 2013; Almeida et al., 2019; Iannitelli et al., 2011; Esfandyari-Manesh et al., 2013; Nallamuthu et al., 2013), which could be explained by the fast dispersion of EOs close to or attached to the surface of the PLGA NPs (Nallamuthu et al., 2013; Hill et al., 2013), as a function of the physicochemical properties of the EOs (Iannitelli et al., 2011). The initial release was followed by a sustained release between 1 and 8 days (Iannitelli et al., 2011; Esfandyari-Manesh et al., 2013; Nallamuthu et al., 2013; Almeida et al., 2019), which can be legitimized by the fact that the EOs must cross the polymeric matrix into the external medium. EOs deeply entrapped in the polymeric matrix have a longer distance to travel and therefore the release rate decreased over time (Esfandyari-Manesh et al., 2013; Hill et al., 2013). The release profile is generally caracterized by the 2-term exponential kinetic model(Gomes et al., 2011), but it can be also close to the Korsmeyer-Peppas model (Almeida et al., 2019).

##### 4.1.5.2. Advantages and drawbacks of PLGA as encapsulating NPs

PLGA presents the advantage to have been recognized as a biocompatible, biodegradable and safe by US FDA and European Medicine Agency (Sharma et al., 2016; Danhier et al., 2012). Moreover, formulations and methods of production are well-described and adapted to various types of substances. PLGA protects EOs from degradation, it allows for their sustained release. Surface properties can be modified to provide stealthiness and/or better interaction with biological materials. There is also the possibility to target PLGA-NPs to specific organs or cells (Danhier et al., 2012). Additionally, PLGA NPs have the ability to cross the blood-brain barrier which make them a suitable polymer used in treating neurological and psychological disorders (Nallamuthu et al., 2013).

Despite all mentioned advantages, PLGA NPs have a relatively low substance loading efficiency. This limits its use as NPs delivery systems in clinical trials (Sharma et al., 2016), in addition to having a high cost of production and difficulty for scale-up (Danhier et al., 2012).

##### 4.1.5.3. Potential applications of PLGA-based-EOs NPs

PLGA-based-EOs NPs were developed to fight against foodborne pathogens such as Gram-positive and Gram-negative bacteria (Hill et al., 2013; Gomes et al., 2011; Nallamuthu et al., 2013). However, some studies have demonstrated that these PLGA-based-EOs NPs were remarkably more effective against Gram + bacteria than against Gram- bacteria (Esfandyari-Manesh et al., 2013). In addition,PLGA-based-EOs NPs were also proposed as a mean against biofilm-associated infections (Iannitelli et al., 2011).

**Figure 2.**
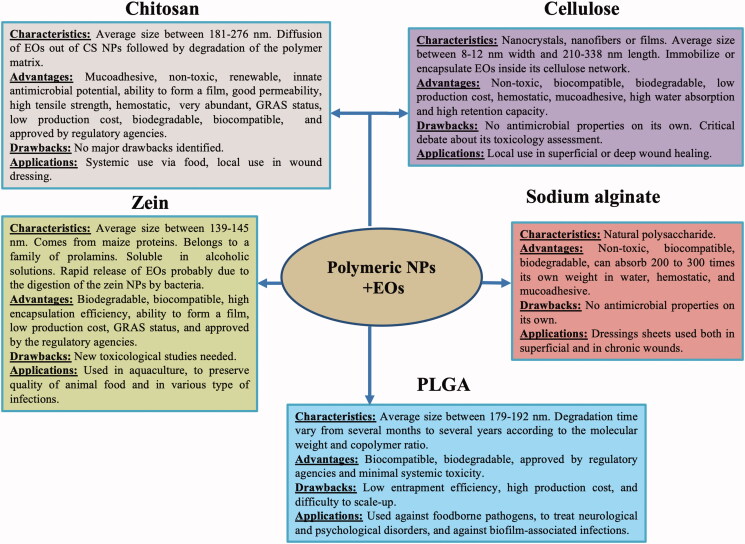
The characteristics, advantages, drawbacks, and applications of polymeric NPs as delivery systems for essential oils.

Figure 2 represents in a synthesized way the characteristics, advantages, drawbacks and applications of polymeric NPs as delivery systems for essential oils.

#### 4.1.6. Lipid-based NPs (solid lipid NPs, nanostructured lipid carriers, nanoemulsions, niosomes, liposomes)

Lipid-based nanoparticles are formed from natural lipids: cocoa butter as solid lipid, and olive oil and sesame oil as liquid lipid (Saporito et al., 2018).To date, several systems based on lipid-based NPs have been described: (a) Solid lipid NPs, which primarily consist of fatty acids or mono-, di-, or triglycerides (Katopodi and Detsi, 2021); (b) Nanostructured lipid carriers, which are emergent generations of lipidic nanoparticles introduced after solid lipid NPs (Khezri et al., 2020); (c) Nanoemulsions, which are transparent and translucent oil in water emulsions with an average droplet diameter of 20 to 200 nm (Naseema et al., 2021); (d) Niosomes, composed of nontoxic self-assembly vesicles, with a single or multiple layered structure, and with the ability to encapsulate hydrophobic and hydrophilic molecules (García-Díaz et al., 2019); (e) Liposomes, which are enclosed spherical vesicles with one or several concentric phospholipidic bilayers and an internal aqueous phase (Sebaaly et al., 2015; Doskocz et al., 2020). Some lipid-based NPs with entrapped EOs (40 to >1000 nm in diameter, mostly polydisperse) were found suitable as antimicrobials in medical or veterinary applications (Table 6).

**Table 6.** Lipid-based-EOs NPs characteristics and potential applications.

Characteristics of lipids used	EOs loaded into lipid-based NPs	NPs size (nm)	NPs polydispersity index	NPs zeta potential (mV)	Methods used	Functionality	Type of study	Medical or veterinary applications	References
Solid lipid NPs and nanostructured lipid carriers (cocoa butter as solid lipid, olive or sesame oil as liquid lipids)	Eucalyptus (*Eucalyptus globulus)* Rosemary (*Rosmarinus officinalis*)	200–300	0.5	−22.07 0.29	High shear homogenization followed by ultrasound application	Physical stability up to 3 months at 2–8 °C	*In vitro* and *in vivo*	Antibacterial activity against *Staphylococcus aureus* ATCC 6538 and *Streptococcus pyogenes* ATCC 19615 (Gram+)	(Saporito et al., 2018)
Solid lipid NPs	Clove *(Eugenia caryophyllata*)	397–1231	0.215–0.680	−15–0.6 to 21.70.2	High-shear homogenization and ultrasound	Physical stability up to 3 months at 2–8 °C	*In vitro*	Antibacterial activity against *Staphylococcus aureus* (Gram+), and against *Salmonella typhi*, *Pseudomonas aeruginosa* (Gram-)	(Fazly Bazzaz et al., 2018)
Nanostructured lipid carriers	Tea tree (*Melaleuca alternifolia*)	150	0.213	−8.69	High pressure homogenization	Improved therapeutic efficacy in *Rhamdia quelen*	*In vivo*	Antibacterial activity against *Pseudomonas aeruginosa* PA01 (Gram-)	(Souza et al., 2017)
Nanostructured lipid carriers	Peppermint (*Mentha piperita*)	40–250	0.4	−10 to −15	Hot melt homogenization	Imporve the healing process of infected wounds in mice by decreasing the tissue bacterial count and edema score	*In vitro* and *in vivo*	*In-vitro* antibacterial activity against *Staphylococcus aureus*, *Staphylococcus epidermidis*, *Bacillus anthracis*, *Staphylococcus pneumonia*, and *Listeria monocytogenes* (Gram+). And against *Escherichia coli*, *Salmonella typhimurium*, *Pseudomonas aeruginosa* (Gram-). *In-vivo* antibacterial activity against *Staphylococcus aureus* (Gram+). And against *Pseudomonas aeruginosa* (Gram-)	(Ghodrati et al., 2019)
Nanostructured lipid carriers	Pennyroyal (*Mentha pulegium)*	40–250	0.4	−10 to −15	Hot melt homogenization	Topical application in mice reduced bacterial count and provoke proliferative phase	*In vitro* and *in vivo*	Antibacterial activity against *Staphylococcus epidermidis* ATCC 12228, *Staphylococcus aureus* ATCC 25923, *Streptococcus pneumoniae* ATCC 49819, *Listeria monocytogenes* ATCC 19133, and *Bacillus anthracis* ATCC 14578 (Gram+). And against *Pseudomonas aeruginosa* ATCC 27853, *Escherichia coli* ATCC 25922 and *Salmonella typhimurium* ATCC 14028 (Gram-)	(Khezri et al., 2020)
Nanoemulsions	Lemongrass (*Cymbopogon flexuosus*) EO	< 200	<0.3	−10.2	Homogenization under high agitation	Greater ability to reduce the adhesion of pathogenic bacteria to the surfaces, inhibiting the biofilm formation	*In vitro*	Antibacterial activity against *Staphylococcus aureus* ATCC 29213 (Gram+). And against *Pseudomonas aeruginosa* PA01 (Gram-)	(da Silva Gündel et al., 2018)
Nanoemulsions	Eucalyptus (*Eucalyptus globulus)* EO	32–142	0.153–0.278	−34.25 to −38.25	Aqueous phase titration	Rapid absorption, improved oral bioavailability, better therapeutic efficacy	*In vivo*	Not reported	(Alam et al., 2018)
Nanoemulsions	Winter savory (*Satureja montana*)	20–200	Not reported	Not reported	Sonication	Improved stability between 32 and 20 °C	*In vitro*	Antimicrobial activity against avian *Escherichia coli* strains (Gram-)	(Rinaldi et al., 2021)
Nanoemulsions	Thyme (*Thymus vulgaris*)	Not reported	Not reported	−25	Sonication	Positive transcriptional modifications of broiler’s digestive enzymes	*In vivo*	Antimicrobial activity against avian *Salmonella enterica* serovar Typhimurium strains (Gram-)	(Ibrahim et al., 2021)
Liposomes Hydrogenated (Phospholipon 80H, Phospholipon 90H) and non-hydrogenated (Lipoid S100) soybean phospholipids were used	Clove *(Eugenia caryophyllata*)	204–380	0.09–0.58	−3 to −38	Ethanol injection, saturated and unsaturated soybean phospholipids, in combination with cholesterol, were used to prepare liposomes	Stability up to 2 months at 4 °C	*In vitro*	Not reported	(Sebaaly et al.,, 2015)

##### 4.1.6.1. Characteristics of the lipid-based NPs and impact on functionality of nanodelivered EOs

The nanostructured lipid carriers are able to penetrate in a bacterial cell, disrupt biomembranes, release polypeptides into the medium and decrease the ATP content of the cell (Ghodrati et al., 2019). The precise *in vivo* mechanism of action of nanodelivered EOs with lipid-based NPs is not known, but the *in vitro* mechanism is known to be related to the interaction with biological membranes (Souza et al., 2017; Ghodrati et al., 2019). This is because the EO, by acting on the biological membranes, causes an expansion of the lipid bilayer, altering the integrity of the membrane, inhibiting the enzymes inside the membrane and thus increasing its fluidity, with the subsequent release of intracellular components (Souza et al., 2017).

More *in vivo* studies have been reported for lipid-based-EOs NPs than for all other nanodelivery systems (Souza et al., 2017; Alam et al., 2018; Ibrahim et al., 2021). Nanoemulsions have been developed for rapid absorption, improved oral bioavailability and better therapeutic efficacy of EOs (Alam et al., 2018). Solid lipid NPs benefit from a physical stability up to 3 months at 2–8 °C (Saporito et al., 2018; Fazly Bazzaz et al., 2018), whereas liposomes have a stability improvement up to 2 months at 4 °C (Sebaaly et al., 2015).

##### 4.1.6.2. Advantages and drawbacks of lipid nanodelivery systems

Lipid nanodelivery systems have the following advantages: (a) appropriate physicochemical properties, (b) good bio-adhesion (due to their flexibility this promotes interaction with the biological substrate and the formation of a bio-adhesive seal, which allows intimate contact between formulation and lesion for good treatment efficiency); (c) good cytocompatibility; (d) improvement of proliferation and wound healing properties toward fibroblasts; (e) synergistic action combined with EOs for antimicrobial properties; (f) efficacy and safety demonstrated *in vivo* (Saporito et al., 2018); (g) administration of lipophilic and hydrophilic substances; (h) increased stability and bioavailability of the substance; (i) safety; (j) good penetration through the lipid bilayer (Ghodrati et al., 2019; Łukawski et al., 2020). Furthermore, solid lipid NPs can (a) transport hydrophobic substances; (b) combine the advantages and reduces the limitations of the use of other colloidal carriers such as nanoemulsions, liposomes, polymeric micro or nanoparticles; (c) they are biodegradable with low systemic toxicity and low cytotoxicity; and (d) it is possible to produce them on a large scale (Fazly Bazzaz et al., 2018). Nanostructured lipid carriers are recognized to be better in terms of loading capacity and stability of the EO contained than other lipid-based nanocarriers such as nanoemulsions or liposomes (Khezri et al., 2020). Nanoemulsion advantages compared to other colloidal vectors of EOs, are: (a) ease of preparation; (b) low cost of preparation; (c) physical and thermodynamic stability; (d) easy formation of nanometric droplet diameters (Alam et al., 2018); and (e) they are safe and nontoxic to animals (Alam et al., 2018). Niosomes are biodegradable, easy to store and handle and have low toxicity (García-Díaz et al., 2019). Moreover, liposomes are biocompatible and non-immunogenic, thus representing an interesting approach to incorporate EOs and to improve their solubility (Sebaaly et al., 2015).

So far, several drawbacks on the use of lipid-based NPs such as solid lipid NPs and nanoemulsions have been identified: (a) poor encapsulation capacity of EOs; (b) limited solubility in water; (c) oxidation of lipids, and (d) release of EOs during storage (Shetta et al., 2019). Although the loading of EOs into liposomes has been studied previously (Sebaaly et al., 2015), the instability of liposomes, rapid release of the entrapped drug, high costs (materials and process) and poor loading efficacy of the drug are major drawbacks to the liposomes which will have to be considered (Hasheminejad et al., 2019; Keawchaoon & Yoksan, 2011).

##### 4.1.6.3. Potential applications of lipid-based-EOs NPs

To date, the use of lipid-based-EOs NPs has been proposed in aquaculture against pathogenic bacteria (Souza et al., 2017) as well as for topical use on chronic wounds and severe burns (Saporito et al., 2018). Nanoemulsions, even at low concentrations, show a great capacity to reduce the adhesion of pathogenic microorganisms to surfaces and to inhibit the formation of biofilm, so this phenomenon explains why they could be used in local treatment (da Silva Gündel et al., 2018).

**Figure 3.**
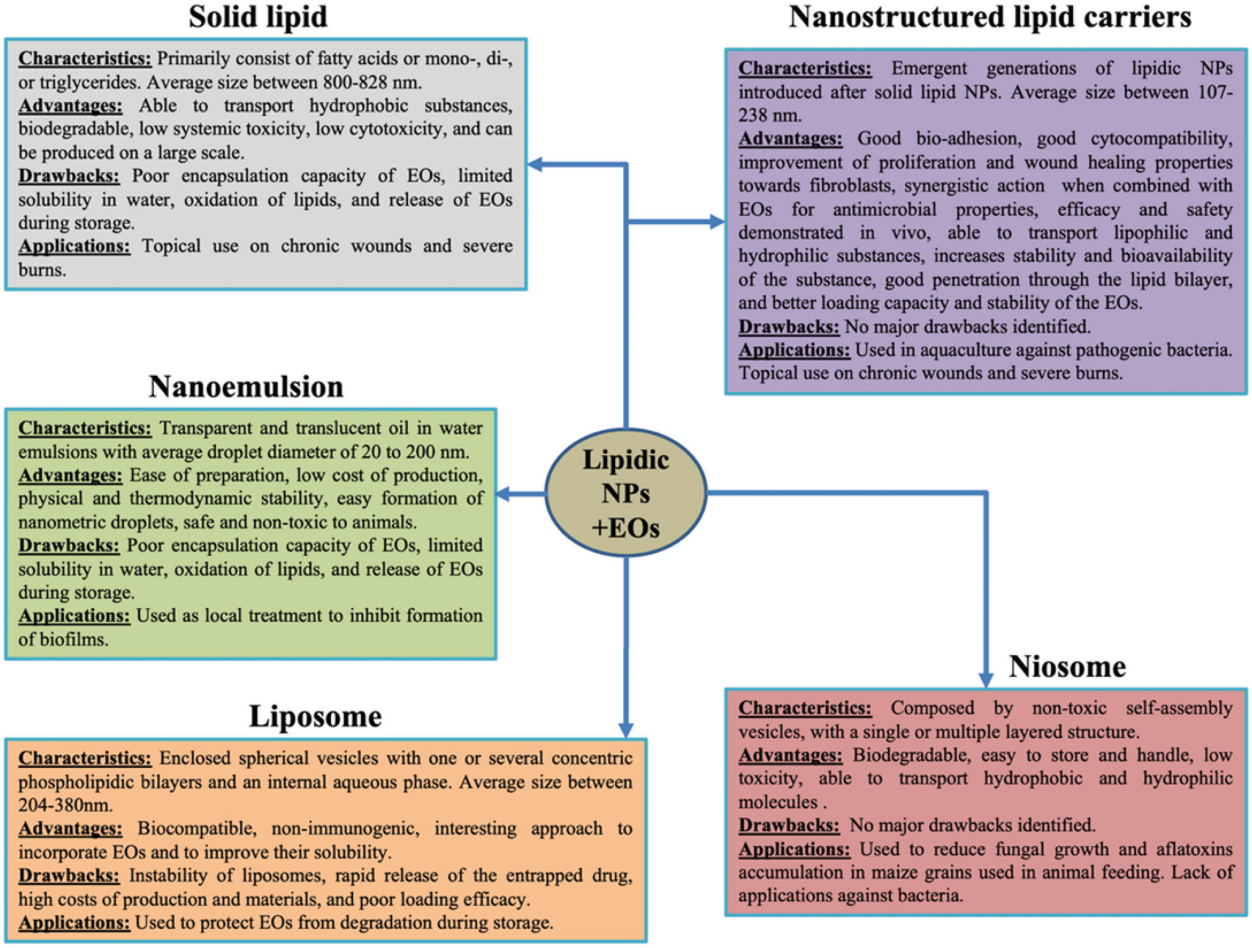
The characteristics, advantages, drawbacks, and applications of lipidic NPs as delivery systems for essential oils.

The characteristics, advantages, drawbacks and main applications of various lipid carriers are summarized in Figure 3.

## 5. Conclusion

In this review, the emphasis was placed on nanodelivery systems loaded with EOs, which turn out to be a promising alternative to fight against antimicrobial resistance in human or in veterinary therapy. Several polymeric and lipidic systems are available for nanodelivery of essential oils with specific advantages of improving EO dispersibility, stability, and release kinetics with an overall improved efficacy over that of free EO. While a wealth of information is available on the topic in the literature as covered in this review, we conclude that more quantitative and *in vivo* studies should be conducted before commercial application of EOs loaded NPs as an alternative to antibiotics, where appropriate.
